# Clinical Risk Factors for Mortality Among Critically Ill Mexican Patients With COVID-19

**DOI:** 10.3389/fmed.2021.699607

**Published:** 2021-08-27

**Authors:** Carmen M. Hernández-Cárdenas, José Alberto Choreño-Parra, Carlos Torruco-Sotelo, Felipe Jurado, Héctor Serna-Secundino, Cristina Aguilar, José G. García-Olazarán, Diana Hernández-García, Eduardo M. Choreño-Parra, Joaquín Zúñiga, Gustavo Lugo-Goytia

**Affiliations:** ^1^Respiratory Intensive Care Unit, Instituto Nacional de Enfermedades Respiratorias Ismael Cosío Villegas, Mexico City, Mexico; ^2^Laboratory of Immunobiology and Genetics, Instituto Nacional de Enfermedades Respiratorias Ismael Cosío Villegas, Mexico City, Mexico; ^3^Escuela Nacional de Ciencias Biológicas, Instituto Politécnico Nacional, Mexico City, Mexico; ^4^Posgrado en Ciencias Biológicas, Universidad Nacional Autónoma de México, Mexico City, Mexico; ^5^Tecnologico de Monterrey, Escuela de Medicina y Ciencias de la Salud, Mexico City, Mexico

**Keywords:** COVID-19, SARS-CoV-2, ARDS, risk factors, mortality

## Abstract

Little literature exists about critically ill patients with coronavirus disease 2019 (COVID-19) from Latin America. Here, we aimed to describe the clinical characteristics and mortality risk factors in mechanically ventilated COVID-19 patients from Mexico. For this purpose, we recruited 67 consecutive mechanically ventilated COVID-19 patients which were grouped according to their clinical outcome (survival vs. death). Clinical risk factors for mortality were identified by machine-learning and logistic regression models. The median age of participants was 42 years and 65% were men. The most common comorbidity observed was obesity (49.2%). Fever was the most frequent symptom of illness (88%), followed by dyspnea (84%). Multilobe ground-glass opacities were observed in 76% of patients by thoracic computed tomography (CT) scan. Fifty-two percent of study participants were ventilated in prone position, and 59% required cardiovascular support with norepinephrine. Furthermore, 49% of participants were coinfected with a second pathogen. Two-thirds of COVID-19 patients developed acute kidney injury (AKIN). The mortality of our cohort was 44.7%. AKIN, uric acid, lactate dehydrogenase (LDH), and a longitudinal increase in the ventilatory ratio were associated with mortality. Baseline PaO2/FiO2 values and a longitudinal recovery of lymphocytes were protective factors against mortality. Our study provides reference data about the clinical phenotype and risk factors for mortality in mechanically ventilated Mexican patients with COVID-19.

## Introduction

The novel severe acute respiratory syndrome coronavirus 2 (SARS-CoV-2), the causative agent of the coronavirus disease 2019 (COVID-19), has rapidly spread worldwide. Although most infected individuals develop mild disease, the spectrum of COVID-19 encompasses severe manifestations that represent up to 5% of cases (1, 2). These forms are characterized by a severe pulmonary inflammation with exudative diffuse alveolar damage and massive capillary congestion accompanied by microthrombi (3, 4). Physiologically, these alterations result in ventilation-perfusion inequalities and severe acute hypoxemic respiratory failure leading to mechanical ventilation (MV) requirement. The exuberant immune response elicited by SARS-CoV-2, together with endothelial dysfunction (5), coagulation disorders (6), and extrapulmonary viral dissemination (7), also precipitate multiorgan failure in a significant proportion of severe COVID-19 cases.

The global case fatality rate (CFR) of COVID-19 varies from 0.2 to 10.5%, depending on several factors, such as age, comorbidities, and geographical region (8). Of note, mortality rates can be as high as 80% among cases admitted to the intensive care unit (ICU) (1). Several clinical and immunological parameters impact on COVID-19-associated morbidity and mortality (2, 9–15). However, most prognostic factors that are currently being used by clinicians have been identified in heterogeneous cohorts of COVID-19 patients with mild to severe manifestations. To what extent those factors independently associated with poor clinical outcomes in the overall population of COVID-19 patients remain informative among individuals in critical condition is not well understood.

The experience with critically ill COVID-19 patients from China, Europe, and the United States has been widely reported in the literature (1, 16–18). However, there is limited information available from Latin America, one of the larger epicenters of the COVID-19 pandemic. Here, we describe the clinical features and outcomes of critically ill COVID-19 patients admitted to the respiratory intensive care unit (RICU) of a national reference center for respiratory diseases in Mexico City. Using a machine-learning algorithm and traditional logistic regression analyses, we also identified clinical risk factors for severe COVID-19-associated mortality. Our results provide reference data about the clinical phenotype of severe COVID-19 among non-Caucasian Hispanic patients from Latin America. Furthermore, our study contributes to a better understanding of the frequency and importance of specific clinical characteristics that determine the risk of mortality in COVID-19 among populations from different geographic regions.

## Methods

### Study Design and Participants

We conducted a prospective cohort study in patients with acute respiratory distress syndrome (ARDS) admitted to the RICU of the Instituto Nacional de Enfermedades Respiratorias Ismael Cosío Villegas (INER) in Mexico City, during the period from March to June of 2020. Individuals that tested positive for SARS-CoV-2 infection in swab samples, bronchial aspirates (BA), or bronchoalveolar lavage (BAL) specimens were eligible. Detection of SARS-CoV-2 was performed by real-time polymerase chain reaction (RT-PCR), as described before (19). None of the participants was coinfected with the human immunodeficiency virus (HIV).

### Data Retrieval and Definitions

Microsoft Excel (MS Excel 365) was used for data collection. Clinical and demographic data were retrieved from patients' medical records, including age, gender, anthropometrics, comorbidities, symptoms, thoracic computed tomography (CT) scan findings, and initial laboratory tests. Initial laboratory tests were defined as the first test results available, typically within 24 h of hospital admission, and included white blood cell counts, liver and kidney function, serum electrolytes, metabolic panel, gasometrical and ventilatory parameters, tissue-injury biomarkers, coagulation parameters, and the severity of disease scores Sequential Organ Failure Assessment (SOFA) and Acute Physiology and Chronic Health disease Classification System II (APACHE-II). Some laboratory parameters, including lymphocyte counts and ventilatory ratio (VR), were monitored continuously, and the last available test results were retrieved for analysis. The primary endpoint of the study was mortality.

ARDS was diagnosed in accordance with the Berlin definition (20). Acute kidney injury (AKIN) was diagnosed in accordance with the Kidney Disease: Improving Global Outcomes (KDIGO) clinical practice guidelines (21). Body mass index (BMI) was calculated as follows: weight (kg)/height (m)^2^. Obesity was defined as a BMI ≥30 kg/m^2^. Bacterial coinfection was defined as a positive culture and consistent clinical data. In cases where the cultures were negative, coinfection was defined based on the presence of persistent fever, leukocytosis, neutrophilia, increased procalcitonin levels, and hemodynamic instability for more than 48 h. Static respiratory-system compliance (Cstat) was calculated as the ratio of the tidal volume to the difference between inspiratory plateau pressure (Pplat) and the positive end-expiratory pressure (PEEP). Driving pressure was calculated as the difference between the Pplat and PEEP. VR was calculated as follows: VR = [minute ventilation (mL/min) × PCO2 (mmHg)]/[predicted body weight × 100 (mL/min) × 37.5 (mmHg)]. Fold changes in variables that were continuously monitored (lymphocyte counts and VR, hereinafter referred to as follow-up parameters) were calculated as the ratio of the difference between values at discharge/death and admission divided by the values of the variables of interest at admission.

### Study Approval

The Institutional Review Board of the INER in Mexico City approved the study. All participants or their legal guardians provided written informed consent in accordance with the Declaration of Helsinki for Human Research. Clinical data were managed according to the Mexican Constitution law NOM-012-SSA3-2012, which establishes the criteria for the execution of clinical investigations in humans.

### Statistical Analysis

Descriptive statistics were used to characterize the study population clinically. Frequencies and proportions were calculated for categorical data. Means, medians, standard deviations (SD), interquartile ranges (IQR), and 95% confidence intervals were used for continuous variables. Differences in categorical variables between groups were assessed by the Fisher exact or Chi-square test. For comparisons of continuous variables, we used the Student T-test or the Mann-Whitney *U*-test, as appropriate. The *K*-means algorithm was used for clustering study participant characteristics according to their clinical outcome (survival or fatality). Before data visualization, clinical features and laboratory parameters were scaled and centered.

All clinical variables were included in a random forest analysis. For this purpose, and considering that the small sample size of the study could impact the performance of the model, 1,000 classification and regression trees (CARTs) were conducted to minimize the prediction error of the analysis measured in terms of Out-of-bag (OOB) error (22). The variables with the highest mean Gini decrease values were considered as having the highest impact on mortality and used as covariates for binomial logistic regression analyses. The accuracy of the selected mortality risk factors identified by random forest and logistic regression models was further evaluated with the area under the Receiver Operating Characteristic (ROC) curve (AUC). In addition, Kaplan–Meier curves were constructed to look for differences in survival according to these variables dichotomized by the ROC curve threshold with the highest diagnostic accuracy estimated using the Youden index. For random forest and logistic regression analyses, patients with any missing value in the variables of interest were excluded. All analyses were conducted using GraphPad Prism 8 (La Jolla, CA) and R Statistical Software (Foundation for Statistical Computing, Vienna, Austria). Specific tests are also mentioned in figure legends. Two-sided *p*-values ≤ 0.05 were considered as significant: ^*^p ≤ 0.05, ^**^p ≤ 0.01, ^***^p ≤ 0.001, ^****^p ≤ 0.0001.

## Results

### Clinical Characteristics of Participants

Data from 67 consecutive COVID-19 patients admitted to the RICU were analyzed. Thirty-seven patients survived, and 30 died (44.7%). Survival rates at different time points after admission are shown in Table 1. Most fatality cases occurred during the second week after RICU admission. The median age of study participants was 42 years (range 23–73), with no differences between survivors and deceased patients (Table 2). Sixty-five percent of participants were men, and the proportion of males was significantly higher in the group of dead patients compared to survivors (80 vs. 54%, *p* < 0.05). Also, the BMI of deceased patients tended to be higher than in survivors. The main comorbidity observed in our study was obesity (49.2%), followed by diabetes (20.8%), and systemic arterial hypertension (SAH; 11.9%). These conditions were similarly distributed across participant groups. Fever was the most frequent manifestation of illness (88%), followed by dyspnea (84%), cough (62%), myalgia (50%), and headache (46%). Only 20% of patients reported diarrhea. The frequency of dyspnea was significantly higher among patients that died.

**Table 1:** Cumulative survival rates in patients with severe COVID-19.

**Time after hospital admission**	**Survival (%)**	**95% CI**
7 days	82.0	70.6–89.4
14 days	56.2	41.6–68.5
21 days	47.6	31.1–62.3
28 days	42.3	25.1–58.5

*Survival rates and their 95% confidence interval (95% CI) were estimated using the Kaplan-Meyer method and the log-rank test*.

**Table 2:** Clinical characteristics of critically ill COVID-19 patients.

**Characteristic**	**Total** ***N*** **= 67**	**Survivors** ***N*** **= 37**	**Deceased** ***N*** **= 30**	***p*** **-value**
Age (years), median (range)	42 (23–73)	37 (23–65)	45 (27–73)	0.2336
Males	44 (65.67)	20 (54.0)	24 (80)	0.0383
BMI	30.5 (26.7–37.5)	29.7 (26.4–34.4)	33.8 (27.4–39.5)	0.0748
**Comorbidities**				
Obesity	33 (49.2)	16 (43.2)	17 (56.6)	0.3302
Diabetes	14 (20.8)	10 (27.0)	4 (13.3)	0.2314
SAH	8 (11.9)	2 (5.4)	6 (20)	0.1264
**Symptoms at onset**				
Fever	44/50 (88)	23/27 (85.1)	21/23 (91.3)	0.674
Cough	31/50 (62)	17/27 (62.9)	14/23 (60.8)	>0.9999
Dyspnea	42/50 (84)	19/27 (70.3)	23/23 (100)	0.005
Myalgia	25/50 (50)	15/27 (55.5)	10/23 (43.4)	0.5709
Headache	23/50 (46)	12/27 (44.4)	11/23 (47.8)	>0.9999
Diarrhea	10/50 (20)	6/27 (22.2)	4/23 (17.3)	0.7356
**CT scan findings**				
Ground glass opacities	38/50 (76)	22/27 (81.4)	16/23 (69.5)	0.5077
Crazing paving pattern	2/50 (4)	2/27 (7.4)	0/23 (0)	0.4931
Consolidation	22/50 (44)	9/27 (33.3)	13/23 (56.5)	0.1532
RICU stay (days)	10 (8–17)	13 (9–18)	8 (6–13)	0.003
**Supportive interventions**				
MV	67 (100)	37 (100)	30 (100)	>0.9999
Prone position	35 (52.2)	17 (45.9)	18 (60)	0.3271
Norepinephrine	40 (59.7)	17 (45.9)	23 (76.6)	0.0133
**Complications**				
AKIN	44 (65.6)	15 (40.5)	29 (96.6)	<0.0001
Stage 1	20 (29.8)	10 (27.0)	10 (33.3)	0.6019
Stage 2	12 (17.9)	1 (2.7)	11 (36.6)	0.0007
Stage 3	12 (17.9)	4 (10.8)	8 (26.6)	0.1165
Coinfection	33 (49.2)	14 (37.8)	19 (63.3)	0.0506

*Data are displayed as n (%), n/N (%), or median (IQR). N is the total number of patients with available data. AKIN, acute kidney injury; BMI, body mass index; CT, computed tomography; IQR, interquartile range; MV, mechanical ventilation; RICU, respiratory intensive care unit; SAH, systemic arterial hypertension. Differences in continuous variables were estimated using the Student T–test or the Mann Whitney U-test. Differences in categorical variables were calculated using the Fisher's exact or the Chi-square test as appropriate*.

Thorax CT scan revealed multilobe ground-glass opacities in 76% of COVID-19 patients, whereas focal consolidations and a crazing-paving pattern were observed in 44% and 4% of participants, respectively. The overall median of days of stay in the RICU was 10 days. All patients required invasive MV, and most of them were intubated within the first 24 h after hospitalization. Fifty-two percent of patients were ventilated in the prone position, and 59% required norepinephrine for cardiovascular support. Of note, a higher proportion of patients that succumbed to COVID-19 required norepinephrine than survivors (76 vs. 45%, *p* < 0.05). Strikingly, up to two-thirds of COVID-19 patients admitted to the RICU developed AKIN, mostly of KDIGO stage 1. However, the percentage of individuals with AKIN was significantly much higher in the group of deceased patients than in survivors (96 vs. 40%, *p* < 0.0001). Furthermore, 49% of participants got coinfected with a second pathogen, and the frequency of coinfection tended to be higher in patients that died of COVID-19 (Table 2).

### Laboratory Parameters of Severely Ill COVID-19 Patients

Most laboratory test results and respiratory parameters at hospital admission were similar in the two groups of COVID-19 patients admitted to the RICU (Table 3). Indeed, in unsupervised clustering analysis, patients with a similar clinical outcome did not cluster together according to their baseline laboratory parameters (Supplementary Figure 1). This finding reflects the high clinical heterogeneity of our entire cohort of severely ill COVID-19 patients admitted to the RICU. Only uric acid, creatinine (Cr), and bilirubin levels, as well as SOFA score, were significantly higher among deceased patients as compared to survivors (Table 3). Procalcitonin levels were also higher in patients that succumbed to the infection than in survivors, but the difference did not reach statistical significance (*p* = 0.0576). In contrast, patients who survived differ significantly from deceased individuals with respect to higher PaO_2_/FiO_2_ values at admission.

**Table 3:** Laboratory parameters of critically ill COVID-19 patients.

**Characteristic**	**Total** ***N*** **= 67**	**Survivors** ***N*** **= 37**	**Deceased** ***N*** **= 30**	***p*** **-value**
**Blood count**				
White blood cells (10^9^/L)	9.4 (7.3–13.03)	9.3 (7.2–12.4)	10.2 (7.3–14)	0.7857
Neutrophils (10^9^/L)	7.9 (5.7–11.4)	7.9 (5.9–10.5)	8.2 (5.3–12.7)	0.9515
Lymphocytes (10^9^/L)	0.8 (0.5–1.02)	0.7 (0.5–1.0)	0.9 (0.6–1.2)	0.1188
NLR	11.2 (6.4–16.2)	11.5 (6.7–17.9)	10.2 (5.6–16)	0.3784
Hgb (g/dL)	13.9 (13.1–15.2)	13.8 (12.7–15.1)	14.0 (13.2–15.4)	0.4139
Platelets (10^9^/L)	243 (188.8–308.8)	252 (202–326.5)	213 (150.8–307)	0.1473
**Metabolic parameters**				
Glucose (mg/dL)	141.2 (108–185)	142.6 (114.5–211)	138.9 (106.5–170.5)	0.4729
Uric acid (mg/dL)	4.0 (2.5–5.0)	3.5 (1.6–4.6)	4.6 (3.6–5.6)	0.0069
Na (mmol/L)	141.2(138.2–143.6)	141 (138.5–143)	141.5(137.8–144.5)	0.4553
K (mmol/L)	4.3 (3.9–4.7)	4.2 (3.9–4.6)	4.3 (3.9–4.7)	0.7808
Total proteins (g/dL)	5.9 (5.5–6.4)	5.8 (5.4–6.3)	6.0 (5.6–6.5)	0.1663
Albumin (g/dL)	2.9 (2.6–3.3)	2.9 (2.6–3.5)	2.9 (2.6–3.2)	0.5667
**Renal function**				
Cr (mg/dL)	0.9 (0.7–1.4)	0.8 (0.6–1.2)	1.2 (0.8–1.8)	0.0105
BUN (mg/dL)	19.6 (13.9–31.5)	19.8 (14.1–28.9)	19.6 (13.7–36.7)	0.3579
**Liver function**				
Total bilirubin (mg/dL)	0.5 (0.4–0–6)	0.4 (0.3–0.5)	0.5 (0.4–0.6)	0.036
AST (U/L)	47.3 (30–76)	42.5 (26.8–69.7)	49.5 (31.9–108.8)	0.1981
ALT (U/L)	39.6 (27.9–67.4)	45.4 (28.1–68.5)	37.4 (25.6–62.6)	0.5368
**Coagulation parameters**				
D dimer (mg/L)	1.0 (0.7–2.1)	1.0 (0.7–2.4)	1.1 (0.7–2.2)	0.6052
INR	1.0 (1.0–1.1)	1.0 (1.0–1.0)	1.0 (0.9–1.1)	0.5146
PT (sec)	15.1 (14.5–16.5)	14.9 (14.6–16.5)	15.4 (14.2–16.5)	0.8317
aPTT (sec)	38.4 (34.6–44.8)	38.5 (35.9–42.1)	37.6 (33.5–49.5)	0.7963
**Other biomarkers**				
LDH (U/L)	494 (357–711)	450 (356.5–589)	566.5 (358.8–738.3)	0.2009
ALP (U/L)	81.6 (64–96.8)	81.8 (59.1–94)	81.6 (66.3–128.6)	0.3252
CPK (U/L)	152.5 (62.3–900)	180.4 (52.2–964)	146.2 (72.5–1016)	0.694
Procalcitonin (ng/mL)	0.16 (0.1–0.37)	0.12 (0.07–0.2)	0.2 (0.1–0.69)	0.0576
**Respiratory parameters**				
SO_2_%	73 (50–85)	73 (41.5–84.5)	71.5 (57.5–85)	0.8582
PCO_2_ (mmHg)	47 (38–56)	49 (41–58)	46 (37.5–52.2)	0.1853
PaO_2_/FiO_2_ (mmHg)	141 (96–177)	158 (110.5–188.5)	121 (88–157.3)	0.0109
DP (cm H2O)	12 (10–14)	12 (10–14.5)	12 (10.6–14.2)	0.4978
Cstat (ml/cm H2O)	33.6 (27–40.9)	33.3 (26.2–40.9)	35.0 (29.6–40.9)	0.4424
VR	2.0 (1.6–2.4)	2.0 (1.7–2.6)	2.0 (1.6–2.3)	0.3483
**Severity of disease**				
SOFA score	5 (4–7)	4 (3–7)	6 (4–8)	0.0155
APACHE-II score	10 (6–17)	9 (5–14)	12 (6–17)	0.2706
**Follow-up parameters**				
Fold change in lymphocytes	0.32 (−0.23–1.12)	0.87 (0.21–1.83)	−0.11 (−0.41–0.68)	<0.0001
Fold change in VR	−0.22 (−0.40–0.22)	−0.35 (−0.45–0.19)	0.15 (−0.23–0.59)	<0.0001

*Data are displayed as n (%) or median (IQR). N is the total number of patients with available data. ALP, alkaline phosphatase; ALT, alanine aminotransferase; APACHE-II, Acute Physiology and Chronic Health disease Classification System II; aPTT, activated partial thromboplastin time; AST, aspartate aminotransferase; Cstat, static compliance; BUN, blood ureic nitrogen; CPK, creatine phosphokinase; Cr, creatinine; DP, driving pressure; FiO2, fraction of inspired oxygen; Hgb, hemoglobin; INR, international normalized ratio; IQR, interquartile range; K, potassium; LDH, lactate dehydrogenase; Na, sodium; NLR, neutrophil/lymphocyte ratio; PaO2, partial pressure of oxygen in arterial blood; PCO_2_, partial pressure of carbon dioxide in the blood; PT, prothrombin time; SO_2_%, oxygen saturation; SOFA, Sequential Organ Failure Assessment; VR, ventilatory ratio. Differences in continuous variables were estimated using the Student T-test or the Mann Whitney U-test. Differences in categorical variables were calculated using the Fisher's exact or the Chi-square test as appropriate. Fold changes were calculated as the ratio of the difference between values at discharge/death and values at admission divided by the initial values of the variables of interest (lymphocyte counts and VR)*.

We also monitored lymphocyte counts and VR in all COVID-19 patients. Remarkably, the group of survivors showed a significant recuperation of lymphocyte counts at discharge from the RICU with respect to the baseline (Table 3). Conversely, deceased patients showed minimal recovery and even displayed depletion of lymphocytes before death. Finally, patients who died showed a significant increase in VR values, whereas survivors were characterized by a decrease in VR at discharge from the RICU. Fold changes in lymphocytes and VR were significantly different between groups (Table 3).

### Mortality Risk Factors in Patients With Severe COVID-19

**Figure 1:**
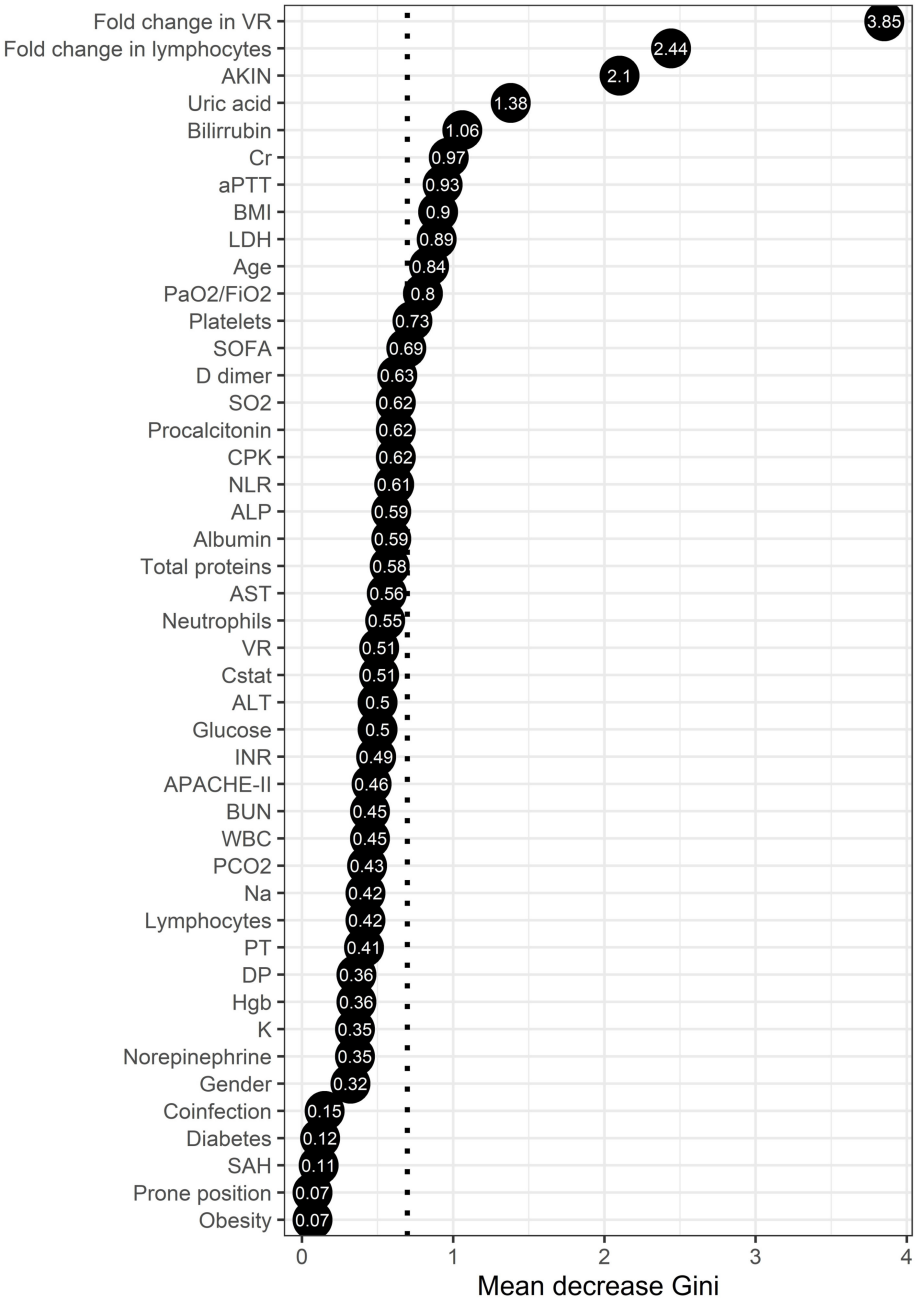
Random forest analysis of the clinical characteristics associated with mortality in critically ill COVID-19 patients. The points represent the mean decrease Gini values, indicative of the importance of each variable to mortality. Variables with mean decease Gini values above the mean importance of the model (discontinuous vertical line) were considered as the most explicative variables of severe COVID-19-associated mortality in our cohort.

We next investigated clinical risk factors for mortality in our cohort of critically ill COVID-19 patients. For this purpose, we performed a random forest analysis using baseline clinical and laboratory characteristics, as well as follow-up parameters of study participants. This is a machine-learning algorithm that accurately estimates the importance of each variable from a dataset for the occurrence of a dichotomous variable (i.e., mortality) (22). The results showed that fold change in VR, fold change in lymphocytes, AKIN, uric acid, bilirubin, Cr, activated partial thromboplastin time (aPTT), BMI, lactate dehydrogenase (LDH), age, and PaO_2_/FiO_2_ were the most explicative variables for mortality, all of them with importance above the overall mean importance of the model (Figure 1).

**Figure 2:**
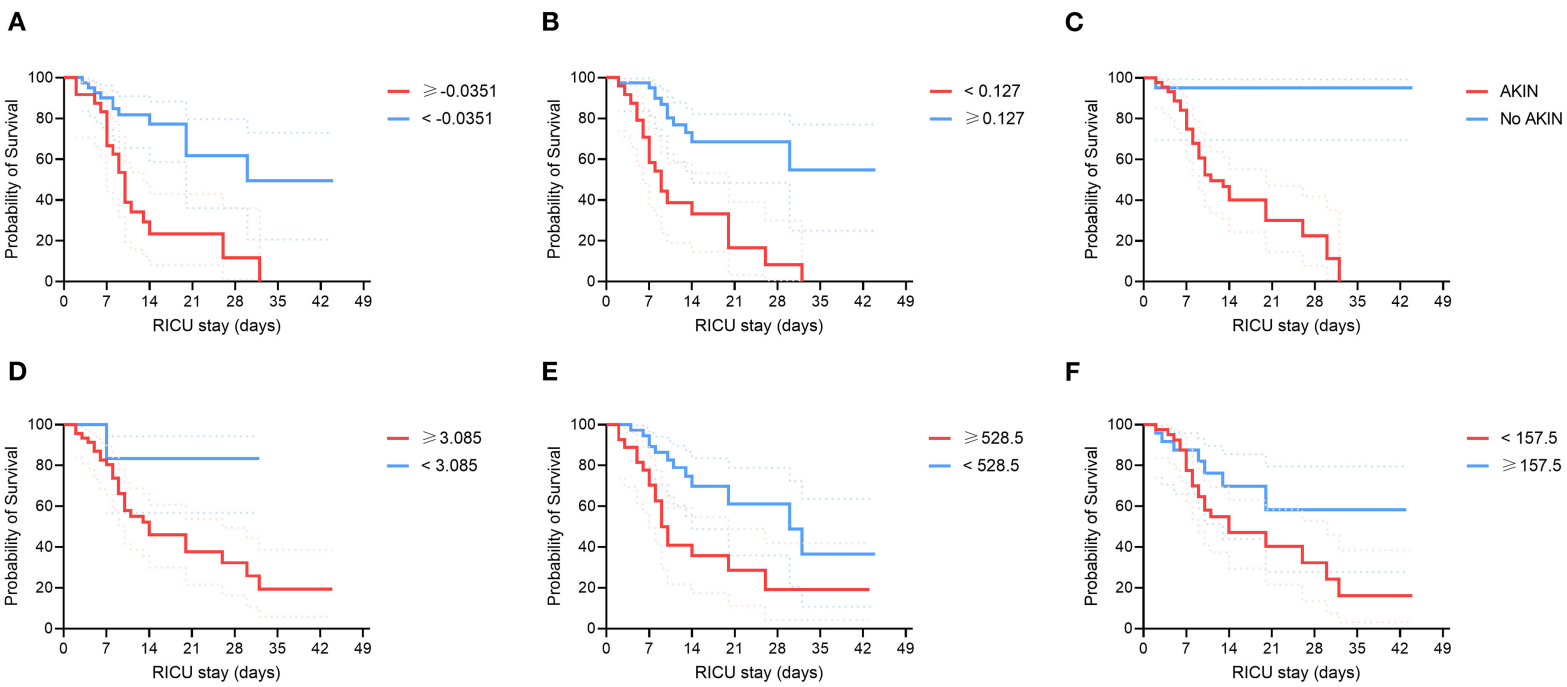
Survival of COVID-19 patients admitted to the RICU according to their clinical characteristics. Critically ill COVID-19 patients were categorized according to different clinical risk factors for mortality identified by random forests and binomial logistic regression analyses. **(A)** Fold change in the ventilatory ratio (VR). **(B)** Fold change in lymphocytes. **(C)** Acute kidney injury (AKIN). **(D)** Uric acid levels. **(E)** Lactate dehydrogenase (LDH) levels. **(F)** PaO_2_/FiO_2_ index. Survival curves were compared using the Kaplan-Meyer method and the log-rank test. Continuous variables were dichotomized using the best ROC curve threshold determined using the Youden index. Fold changes in lymphocyte counts and VR are defined as the ratio of the difference between values at discharge/death and values at admission divided by the initial values of these variables.

From these variables identified in the random forest analysis, only fold change in VR, AKIN, uric acid, and LDH were independent mortality risk factors in binomial logistic regression analyses. Meanwhile, baseline PaO_2_/FiO_2_ values and fold change in lymphocytes were protective factors (Table 4). In fact, patients with an increase in VR (fold change ≥-0.0351 from baseline), AKIN, high uric acid (≥3.085 mg/dL), and elevated LDH levels (≥528.5 U/L), showed significantly lower survival rates at 28 days after admission to the RICU (Table 5; Figure 2). In contrast, patients with a longitudinal increase in lymphocyte counts (fold change ≥0.127 from baseline) and higher PaO2/FiO2 (≥157.5) values at admission had lower mortality rates.

**Table 4:** Logistic regression analysis of risk factors for COVID-19-associated mortality.

**Variable**	**OR**	**95% CI**	***p*** **-value**
Fold change in VR	50.20	8.187–548.3	<0.0001
Fold change in lymphocytes	0.226	0.08815–0.4747	<0.0001
AKIN	36.73	6.631–692.1	<0.0001
Uric acid (mg/dL)	1.237	1.003–1.587	0.0463
Total bilirubin (mg/dL)	3.533	0.3235–45.64	0.3023
Cr (mg/dL)	1.086	0.7491–1.613	0.6568
aPTT (sec)	1.040	0.9702–1.120	0.2696
BMI	1.068	0.9930–1.162	0.0778
LDH (U/L)	1.002	1.000–1.005	0.0360
Age (years)	1.019	0.9778–1.063	0.3768
PaO_2_/FiO_2_ (mmHg)	0.9872	0.9751–0.9984	0.0241

*95% CI, 95% confidence interval; AKIN, acute kidney injury; aPTT, activated partial thromboplastin time; BMI, body mass index; Cr, creatinine; FiO_2_, fraction of inspired oxygen; LDH, lactate dehydrogenase; OR, odds ratio; PaO_2_, partial pressure of oxygen in arterial blood; VR, ventilatory ratio. Fold changes in lymphocyte counts and VR are defined as the ratio of the difference between values at discharge/death and values at admission divided by the initial values of these variables*.

**Table 5:** Survival rates of severely ill COVID-19 patients according to their clinical characteristics.

**Variable**	**ROC** **AUC (95% CI)**	**Sensitivity (%)**	**Specificity (%)**	**Survival** **(%, 95% CI)**	***p*** **-value**
Fold change in VR	0.8441 (0.7521–0.9361)	63.33	88.24		<0.0001
≥-0.0351				11.68 (1.09–36.03)	
<-0.0351				61.82 (36.03–79.72)	
Fold change in lymphocytes	0.8039 (0.6981–0-9098)	63.33	85.29		<0.0001
≥0.127				68.51 (48.44–82.09)	
<0.127				8.31 (0.58–29.97)	
AKIN	N/A	96.67	59.46		<0.0001
Yes				22.53 (7.84–41.79)	
No				95.00 (69.47–99.28)	
Uric acid (mg/dL)	0.6971 (0.5682–0.8260)	90	44.12		0.025
≥3.085				32.29 (16.25–49.49)	
<3.085				83.33 (56.76–94.29)	
LDH (U/L)	0.6245 (0.4822–0.7668)	60	73.53		0.0035
≥528.5				19.10 (4.30–41.85)	
<528.5				61.09 (35.88–78.90)	
PaO_2_/FiO_2_ (mmHg)	0.6569 (0.5232–0.7905)	76.67	50		0.0706
≥157.5				58.18 (27.79–79.53)	
<157.5				32.28 (13.51–52.78)	

*95% CI, 95% confidence interval; AKIN, acute kidney injury; AUC, area under the curve; FiO_2_, fraction of inspired oxygen; LDH, lactate dehydrogenase; N/A, not applicable; PaO_2_, partial pressure of oxygen in arterial blood; ROC, receiver operating characteristic curve; VR, ventilatory ratio. Survival rates at 28 days of admission, and their 95% CI were estimated using the Kaplan-Meyer method and the log-rank test. Best ROC curve thresholds were calculated with the Youden index. Sensitivity and specificity for AKIN were calculated by the Wilson/Brown method. Fold changes in lymphocyte counts, and VR are defined as the ratio of the difference between values at discharge/death and values at admission divided by the initial values of these variables*.

Finally, a binomial regression analysis of the variables not identified as important for mortality by random forest analysis showed that male gender, use of norepinephrine, and SOFA score were independently associated with mortality. In contrast, the neutrophil to lymphocyte ratio (NLR) was a protective factor (Supplementary Table 1). From these, only norepinephrine usage was associated with significantly lower survival rates in the Kaplan-Meier and log-rank test analysis (Supplementary Figure 2). However, in a second random forest model using all the independent mortality risk and protective factors identified only by binomial logistic regression, none of these additional factors showed to be explicative for mortality (Supplementary Figure 3).

## Discussion

In the current study, we report the clinical characteristics of a cohort of critically ill patients with COVID-19 that were admitted to the RICU of a national reference center for respiratory diseases in Mexico City. Our analyses showed that most demographic, clinical, radiological, and biochemical characteristics of Mexican patients with severe SARS-CoV-2 infection resemble those reported previously by other groups from China, Europe, and the United States. Furthermore, we determined which factors were independently associated with mortality using a non-conventional statistical approach that included machine-learning algorithms and traditional regression analyses. This strategy of analysis allowed us to identify six variables that had the highest impact on the mortality of our cohort: fold change in VR, fold change in lymphocyte counts, AKIN, uric acid, LDH, and PaO^2^/FiO^2^. From these, fold change in lymphocytes and PaO^2^/FiO^2^ at admission acted as independent protective factors.

A dramatic depletion of total lymphocytes, as well as of CD8+ and CD4+ T-cells, has been reported in patients with SARS-CoV-2 infection (23–25). This phenomenon is the expression of a dysregulated immune response elicited by the virus that favors immunosuppression and is associated with a high risk of secondary bacterial infection, septic shock, and organ dysfunction (26). Indeed, lymphopenia has been described as a marker of severity and a predictor of mortality in COVID-19 (27). In our cohort, baseline lymphocyte counts were extremely low in all patients, with no differences between survivors and non-survivors. These data may indicate that despite lymphopenia is a readout of severity in the overall population of COVID-19 patients, this marker is not further informative when used only among critically ill individuals. Thus, lymphocyte counts on admission should be used only in the decision-making for patients with mild-to-moderate forms of the disease to predict the progression to severe COVID-19.

Recovery of the adaptive immune system with an increase in the number of circulating T lymphocytes is necessary to eliminate the virus (28). Notably, we also found that longitudinal increases in the number of circulating lymphocytes (here expressed as a fold change in lymphocytes) have a strong protective effect against mortality. In other words, a longitudinal increase in lymphocytes associates with a decreased mortality risk, while a decrease in lymphocytes correlates with a significant increase in the risk of death. This result is consistent with the rapid and dramatic restoration of peripheral T lymphocytes observed in patients who recovered from SARS-CoV-2 infection (28). Hence, our results indicate that changes in the lymphocyte counts could be used as a parameter to guide therapeutic decisions for critically ill COVID-19 patients. For instance, this parameter could determine which patients would benefit from steroids use. These drugs could have both favorable and unfavorable consequences. For example, in patients with an exaggerated inflammatory response, steroids may reduce organ damage. In contrast, in patients with severe immunosuppression, steroids could accentuate this defect, increasing the risk of sepsis and mortality; applying treatments that stimulate the immune system could be useful in these patients (29).

VR is governed by the production of carbon dioxide (CO_2_) and the ventilatory efficiency (1-(Vd/Ve)) and can be easily calculated at the patient's bedside using ventilation and blood gas parameters. It correlates with the percentage of dead space and is also associated with an increased risk of mortality (30–32). Previous studies on patients with ARDS and COVID-19 have reported a significant association between the VR at admission and mortality (33). In our patients, we did not observe this association upon admission to the RICU. However, we observed that a longitudinal increase in VR was a marker of poor clinical outcome in our cohort of patients with ARDS due to COVID-19. This result is consistent with other reports that demonstrated that an increase in the fraction of dead space during the first weeks of ARDS is an independent predictor of mortality (30–32). In summary, the worsening of the VR in our cohort was independently associated with an increased risk of mortality. Similar to the tidal volume adjusted for the predicted weight, plateau pressure, DP, and PaO2/FiO2 ratio, the VR should be monitored daily and used to make adjustments to the ventilatory parameters, always taking into account the variables mentioned above.

The most striking mortality risk factor identified in our study population was the incidence of AKIN. The injury of the kidney has been widely reported in patients with sepsis and severe ARDS associated with other respiratory pathogens, such as influenza viruses (34–36). Indeed, AKIN is a well-recognized mortality risk factor in patients with severe pneumonia caused by the pandemic influenza A(H1N1)pdm09 virus (37). Similarly, a high incidence of AKIN has been reported in patients with COVID-19. For instance, Hirsch et al. reported an incidence of 36.6% in a cohort of 5,449 patients with COVID-19 (38). However, in patients with respiratory failure who required invasive MV, the incidence of AKIN was 89.7%, and in those who required hemodialysis the mortality was 55% (38). In our cohort, up to 65% of the patients developed AKIN, and its incidence had a strong effect on mortality. Several mechanisms could contribute to the development of AKIN among patients with severe COVID-19, including direct injury driven by the virus and detrimental effects of the high levels of circulating proinflammatory mediators, endothelial dysfunction, and micro-thrombosis of renal blood vessels. Independently of the causative mechanism, the implementation of the KDIGO supportive care guidelines to avoid AKIN or to prevent progression to more advanced stages must be a priority in critically ill patients with COVID-19 (21).

Uric acid, LDH, and PaO_2_/FiO_2_ also impacted on mortality of our study population. Interestingly, little evidence exists about the prognostic value of uric acid in COVID-19. Hence, ours is among the first studies that bring forward this marker for mortality prediction after SARS-CoV-2 infection. As uric acid levels primarily depend on the balance between its production and excretion through the urine, we speculate that the elevated uric acid levels observed among critically ill COVID-19 that died are related to the high incidence of renal dysfunction in these individuals. Notably, other biomarkers of renal function, such as Cr, were not associated with mortality. Collectively, these data indicate that uric acid may be a more useful readout of the renal function status than Cr and blood ureic nitrogen (BUN) among patients with COVID-19 in critical conditions. Regarding LDH, several studies have reported that this is a good marker to predict mortality in patients infected with SARS-CoV-2 (39, 40). Hence, our study reinforces the usage of LDH as a prognostic indicator of mortality that could be useful to guide therapeutic interventions.

Finally, the PaO2/FiO2 ratio showed a significant protective effect against mortality in our analyses. The PaO2/FiO2 ratio is a crucial determinant of the severity of ARDS, according to the Berlin definition (20). The majority of our patients showed ground-glass opacities on chest tomography, without extensive consolidation images. This explains the rapid response of many patients to oxygen administration and the poor response at this stage to recruitment maneuvers because there are no extensive recruitable consolidation areas. Therefore, the primary mechanism of hypoxemia in these patients in the initial phase is an abnormality in the distribution between ventilation and blood flow; the latter is assumed to be abnormal due to endothelial and vascular alterations documented among COVID-19 patients (41). Therefore, the PaO2/FiO2 ratio may be a good physiological biomarker of the amount of pulmonary shunt and lung parenchymal damage in the early phase of ARDS due to COVID-19, which explains why this parameter was a protective factor against mortality in our study. In contrast with our results, other investigations have shown that the PaO2/FiO2 ratio is not a strong predictor of mortality, which may be related to the clinical heterogeneity observed in studies involving patients with moderate-to-severe COVID-19.

One limitation of the study is the small size of the cohort, which originated from a single third-level center in Mexico City. Therefore, although our results are consistent with those reported in China, Europe, and North America, and despite the machine-learning approach used in our study may compensate for this caveat, the predictive value of the mortality risk factors identified here require further external validations in larger cohorts. In summary, we described the clinical characteristics of a cohort of critically ill Mexican patients with COVID-19 and identified independent factors associated with mortality. Based on our results, it is possible to suggest some management recommendations in patients with COVID-19 who require intensive care. These include respiratory management based on low tidal volumes and adjustment of parameters according to the VR. Measures to protect kidney function and adjustment of fluid balance according to volume responsiveness is also recommendable. Furthermore, the avoidance of immunosuppressants in patients who do not show lymphocyte recovery, strict measures to prevent nosocomial infections, early detection, and aggressive treatment of suspected coinfections are crucial interventions. These simple measures could reduce the risk of mortality until an effective therapy against SARS-CoV-2 infection is available.

## Data Availability Statement

The raw data supporting the conclusions of this article will be made available by the authors, without undue reservation.

## Ethics Statement

The studies involving human participants were reviewed and approved by the Institutional Review Board of the Instituto Nacional de Enfermedades Respiratorias Ismael Cosío Villegas in Mexico City under the approval number B09-20. The patients/participants provided their written informed consent to participate in this study.

## Author Contributions

CH-C, JC-P, JZ, and GL-G: designed the research study. CH-C, CT-S, FJ, HS-S, CA, JG-O, DH-G, and GL-G: recruited patients. CH-C, JC-P, CT-S, FJ, HS-S, CA, JG-O, DH-G, and GL-G: retrieved clinical data. JC-P and EC-P: performed statistical analyses of the data. JZ: discussed the manuscript. JC-P, JZ, CH-C, and GL-G: drafted the manuscript. All authors contributed to the article and approved the submitted version.

## Conflict of Interest

The authors declare that the research was conducted in the absence of any commercial or financial relationships that could be construed as a potential conflict of interest.

## Publisher's Note

All claims expressed in this article are solely those of the authors and do not necessarily represent those of their affiliated organizations, or those of the publisher, the editors and the reviewers. Any product that may be evaluated in this article, or claim that may be made by its manufacturer, is not guaranteed or endorsed by the publisher.
