# Supplementary material for: Clinical Risk Factors for Mortality Among Critically Ill Mexican Patients With COVID-19
Source: Front Med (Lausanne). 2021 Aug 27;8:699607. doi: 10.3389/fmed.2021.699607 (PMC8429783; doi:10.3389/fmed.2021.699607)
Supplement: Supplementary file 1 [file Data_Sheet_1.docx]

Supplementary Material

# Supplementary Tables

| Table S1. Logistic regression analysis of risk factors for COVID-19-associated mortality | | | |
| --- | --- | --- | --- |
| Variable | **Univariate OR** | **95% CI** | ***p-*value** |
| Male gender | 3.556 | 1.205 – 11.62 | 0.0210 |
| Obesity | 1.868 | 0.6966 – 5.139 | 0.2151 |
| Diabetes | 0.3692 | 0.09141 – 1.265 | 0.1148 |
| SAH | 4.000 | 0.8376 – 28.96 | 0.0838 |
| Prone position | 1.500 | 0.5583 – 4.110 | 0.4220 |
| Norepinephrine | 3.696 | 1.294 – 11.47 | 0.0141 |
| Coinfection | 2.468 | 0.9125 – 6.941 | 0.0754 |
| White blood cells (10^9^/L) | 1.038 | 0.9268 – 1.167 | 0.5192 |
| Neutrophils (10^9^/L) | 1.025 | 0.9099 – 1.157 | 0.6833 |
| Lymphocytes (10^9^/L) | 2.796 | 0.9485 – 10.06 | 0.0629 |
| NLR | 0.9475 | 0.8761 – 0.9989 | 0.0436 |
| Hgb (g/dL) | 1.059 | 0.8244 – 1.377 | 0.6525 |
| Platelets (10^9^/L) | 0.9985 | 0.9933 – 1.003 | 0.5482 |
| Glucose (mg/dL) | 0.9980 | 0.9893 – 1.006 | 0.6428 |
| Na (mmol/L) | 1.039 | 0.9190 – 1.180 | 0.5424 |
| K (mmol/L) | 1.164 | 0.5374 – 2.596 | 0.6958 |
| Total proteins (g/dL) | 1.868 | 0.8080 – 4.582 | 0.1453 |
| Albumin (g/dL) | 0.6553 | 0.2451 – 1.548 | 0.3422 |
| BUN (mg/dL) | 1.007 | 0.9881 – 1.029 | 0.4759 |
| AST (U/L) | 1.005 | 0.9952 – 1.015 | 0.3400 |
| ALT (U/L) | 1.003 | 0.9925 – 1.015 | 0.5485 |
| D dimer (mg/L) | 0.9523 | 0.8068 – 1.053 | 0.3677 |
| INR | 3.656 | 0.01540 – 1026 | 0.6399 |
| PT (sec) | 0.9866 | 0.6924 – 1.401 | 0.9390 |
| ALP (U/L) | 1.009 | 0.9992 – 1.023 | 0.0742 |
| CPK (U/L) | 1.000 | 0.9999 – 1.001 | 0.1524 |
| Procalcitonin (ng/mL) | 1.221 | 0.7334 – 2.157 | 0.4418 |
| SO_2_% | 1.008 | 0.9837 – 1.035 | 0.5099 |
| PCO_2_ (mmHg) | 0.9743 | 0.9272 – 1.021 | 0.2780 |
| DP (cm H2O) | 1.044 | 0.8884 – 1.232 | 0.5991 |
| Cstat (ml/cm H2O) | 1.006 | 0.9570 – 1.059 | 0.8002 |
| VR | 0.7156 | 0.3202 – 1.514 | 0.3840 |
| SOFA score | 1.314 | 1.030 – 1.711 | 0.0274 |
| APACHE-II score | 1.042 | 0.9630 – 1.130 | 0.3085 |
| 95% CI, 95% confidence interval; ALP, alkaline phosphatase; ALT, alanine aminotransferase; APACHE-II, Acute Physiology and Chronic Health disease Classification System II; AST, aspartate aminotransferase; Cstat, static compliance; BUN, blood ureic nitrogen; CPK, creatine phosphokinase; DP, driving pressure; Hgb, hemoglobin; INR, international normalized ratio; NLR, neutrophil/lymphocyte ratio; OR, odds ratio; PCO_2_, partial pressure of carbon dioxide in the blood; PT, prothrombin time; SAH, systemic arterial hypertension; SO_2_%, oxygen saturation; SOFA, Sequential Organ Failure Assessment; VR, ventilatory ratio. | | | |

# Supplementary Figures


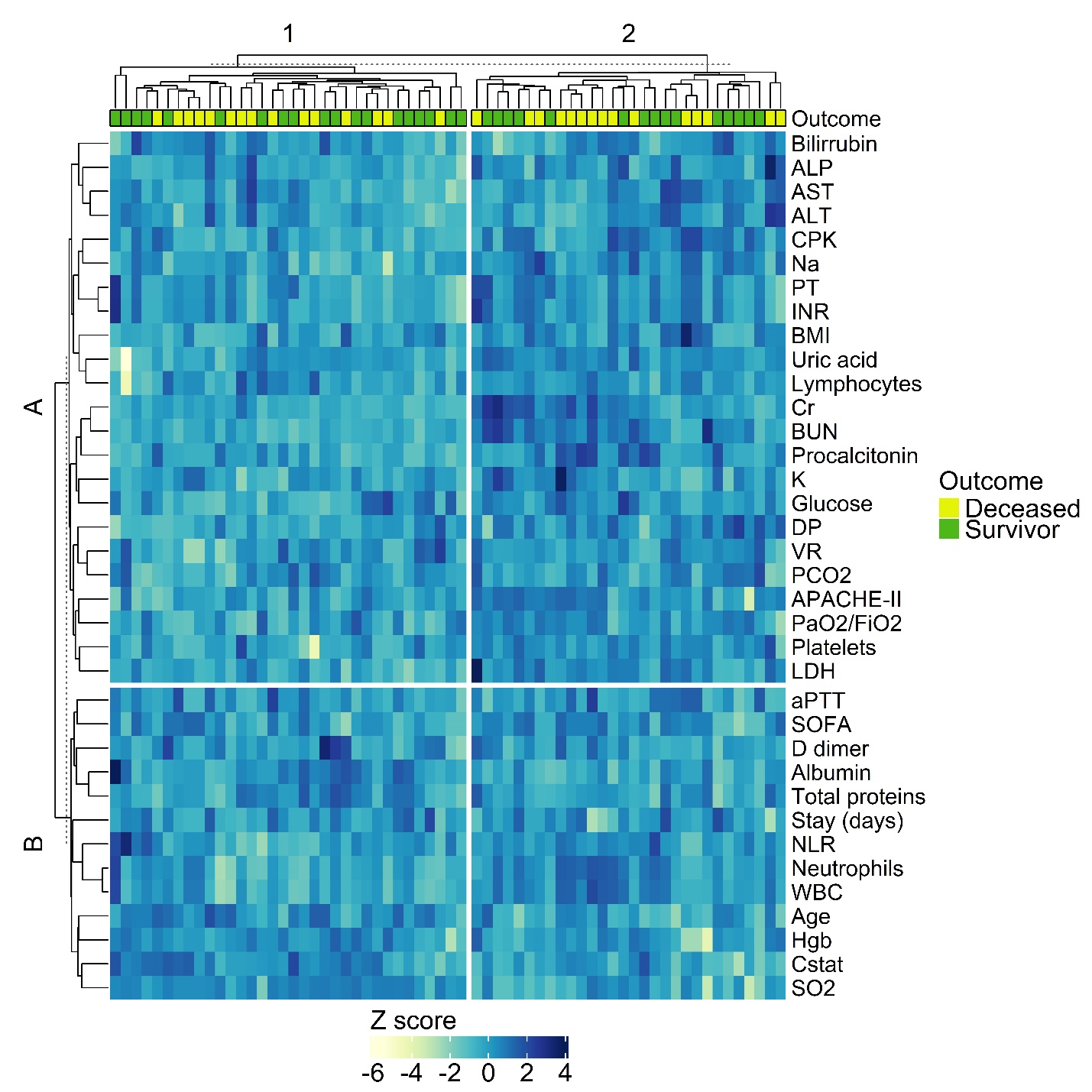


**Fig. S1.** K-means clustering algorithm of the clinical and laboratory characteristics of COVID-19 patients in critical condition. Before data visualization, clinical features and laboratory parameters were scaled and centered. Patients with any missing value in the variables of interest were excluded.


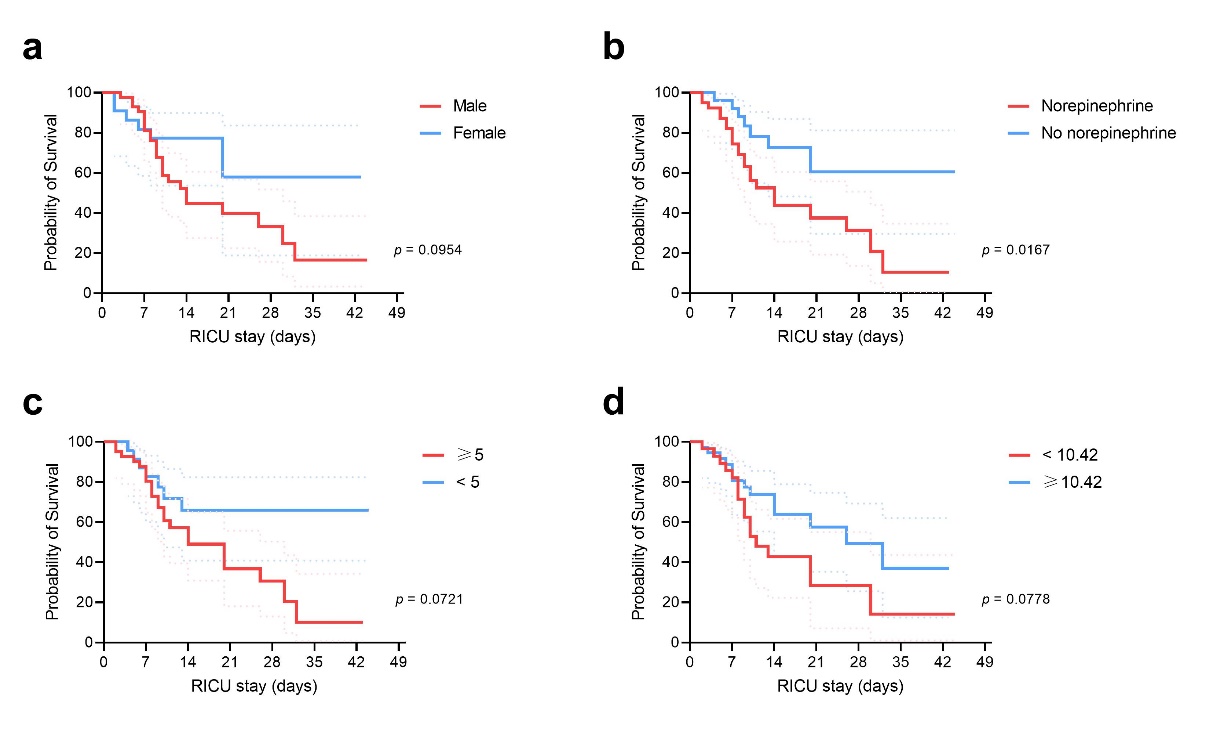


**Fig. S2. Survival of COVID-19 patients admitted to the RICU according to their clinical characteristics.** Critically ill COVID-19 patients were categorized according to different clinical risk factors for mortality identified by binomial logistic regression analyses. (a) Gender. (b) Use of norepinephrine. (c) Sequential Organ Failure Assessment score. (d) Neutrophil to lymphocyte ratio. Survival curves were compared using the Kaplan-Meyer method and the log-rank test. Continuous variables were dichotomized using the best ROC curve threshold determined using the Youden index.


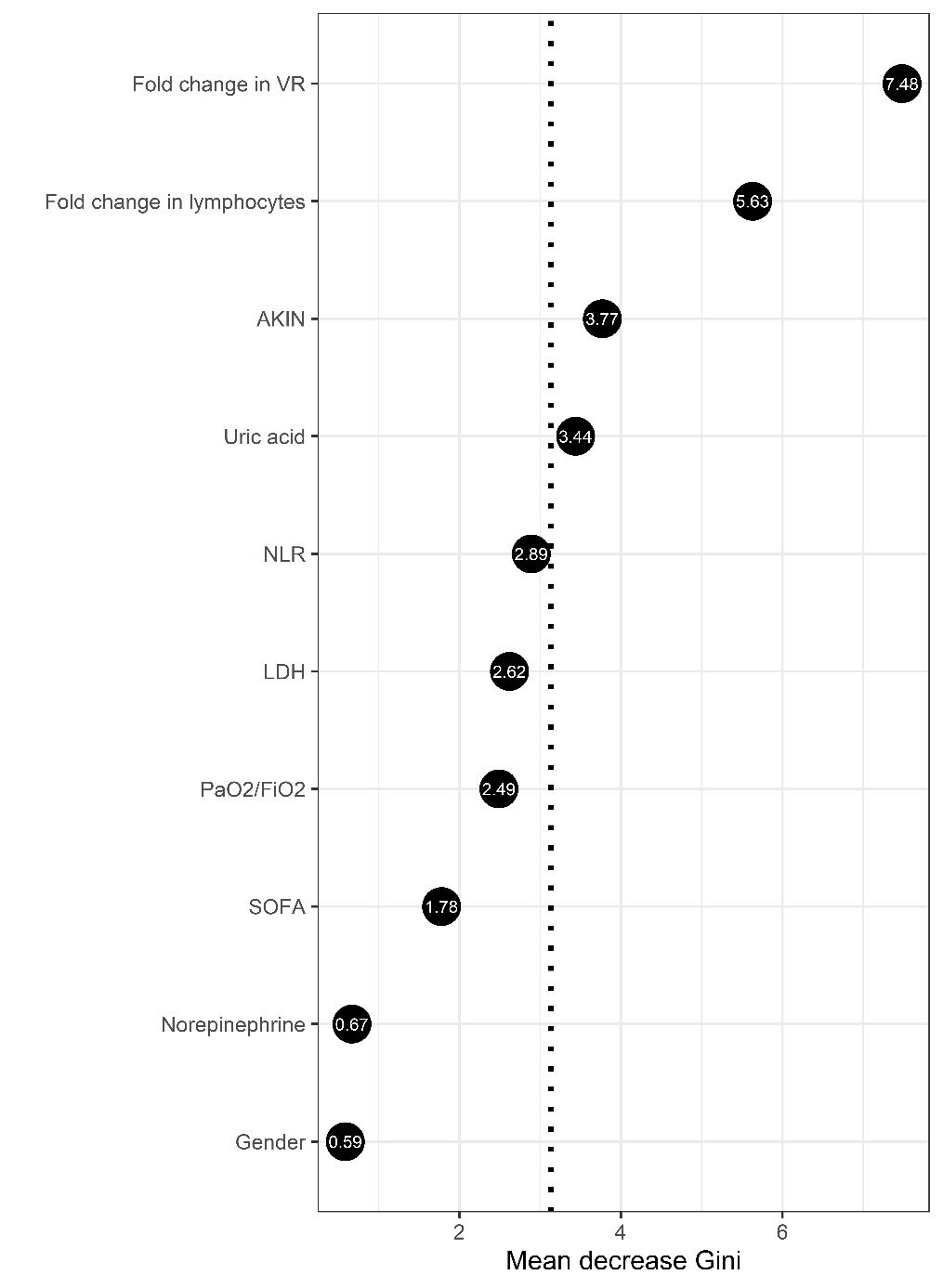


**Figure S3. Random forest analysis of selected clinical characteristics associated with mortality in critically ill COVID-19 patients.** All variables with statistically significant association with mortality in the binomial regression analyses were included in a random forest analysis. The points represent the mean decrease Gini values, indicative of the importance of each variable to mortality. Variables with mean decease Gini values above the mean importance of the model (discontinuous vertical line) were considered as the most explicative variables of severe COVID-19-associated mortality in our cohort.
